# Faecal Microbiota in Infants and Young Children with Functional Gastrointestinal Disorders: A Systematic Review

**DOI:** 10.3390/nu14050974

**Published:** 2022-02-25

**Authors:** Denise Hofman, Urszula Kudla, Mohamad Miqdady, Thi Viet Ha Nguyen, Sofía Morán-Ramos, Yvan Vandenplas

**Affiliations:** 1 FrieslandCampina, Stationsplein 1, 3818 LE Amersfoort, The Netherlands; urszula.kudla@frieslandcampina.com; 2 Ped. GI, Hepatology & Nutrition Division, Sheikh Khalifa Medical City, P.O. Box 51900, Abu Dhabi 51133, United Arab Emirates; msmiqdady@yahoo.com; 3 Department of Pediatrics, Hanoi Medical University, Hanoi 116001, Vietnam; vietha@hmu.edu.vn; 4 Unidad de Genomica de Poblaciones, Instituto Nacional de Medicina Genomica, Mexico City 14610, Mexico; smoran@inmegen.gob.mx; 5 Paediatric Gastro-Enterology and Nutrition, Vrije Universiteit Brussel, UZ Brussel, KidZ Health Castle, 1050 Brussels, Belgium; yvan.vandenplas@uzbrussel.be

**Keywords:** faecal microbiota, functional gastrointestinal disorders (FGIDs), colic, functional constipation

## Abstract

Functional gastrointestinal disorders (FGIDs) refer to gastrointestinal tract issues that lack clear structural or biochemical causes. Their pathophysiology is still unclear, but gut microbiota alterations are thought to play an important role. This systematic review aimed to provide a comprehensive overview of the faecal microbiota of infants and young children with FGIDs compared to healthy controls. A systematic search and screening of the literature resulted in the inclusion of thirteen full texts. Most papers reported on infantile colic, only one studied functional constipation. Despite methodological limitations, data show alterations in microbial diversity, stability, and colonisation patterns in colicky infants compared to healthy controls. Several studies (eight) reported increases in species of (pathogenic) Proteobacteria, and some studies (six) reported a decrease in (beneficial) bacteria such as Lactobacilli and Bifidobacteria. In addition, accumulation of related metabolites, as well as low-grade inflammation, might play a role in the pathophysiology of infantile colic. Infants and toddlers with functional constipation had significantly lower levels of Lactobacilli in their stools compared to controls. Microbial dysbiosis and related changes in metabolites may be inherent to FGIDs. There is a need for more standardised methods within research of faecal microbiota in FGIDs to obtain a more comprehensive picture and understanding of infant and childhood FGIDs.

## 1. Introduction

Functional gastrointestinal disorders (FGIDs) refer to a wide range of gastrointestinal (GI) tract disorders that cannot be explained by structural or biochemical abnormalities [1]. Approximately 50% of all infants are estimated to suffer from at least one FGID during their first months of life [2]. The main FGIDs in infants and toddlers are infantile colic, regurgitation or gastroesophageal reflux (GER), functional constipation, functional diarrhoea, cyclic vomiting syndrome, infant dyschezia, and infant rumination syndrome [1]. GER is the most prevalent infantile FGID and is defined as the passage of gastric contents into the oesophagus, with or without regurgitation and/or vomiting [1]. According to the Rome IV criteria, infantile colic typically presents as recurrent and prolonged periods of infant crying, fussing, or irritability reported by caregivers that occur without obvious cause and cannot be prevented or resolved by caregivers, in absence of failure to thrive, fever, or illness. “Fussing” refers to intermittent distressed vocalisation and has been defined as “behaviour that is not quite crying but not awake and content either.” Infants often fluctuate between crying and fussing, so that the two symptoms are difficult to distinguish in practice [1]. It is typically assessed by the frequency and duration of crying time and historically, it was defined as excessive crying for more than 3 h per day, for at least 3 days per week, during more than 3 weeks in an otherwise healthy baby, more commonly known as “the rule of 3′s” [3]. 

With the exception of functional constipation, most FGIDs are transient; they disappear spontaneously over time, usually within the first year of life [1]. However, FGIDs can have both short- and long-term effects on health and quality of life of infants and their caregivers. Short-term consequences include feeding difficulties, discontinuation of breastfeeding, and parental stress. Longer-term effects include behavioural and sleep problems, and an increased risk of developing FGIDs later in life, as well as childhood migraine, asthma, or atopic disease [4,5,6]. Although functional disorders are typically discussed separately, most infants present with a combination of different FGIDs [7]. The presence of multiple FGIDs is more likely to be associated with a decreased quality of life compared to single FGIDs [8].

The pathophysiology of FGIDs is still unclear. Underlying mechanisms are considered to be multifactorial [9]. In addition to type of feeding, feeding behaviours, and neurodevelopmental and psychological factors, mechanisms related to the GI tract, such as gas formation, motility, gut inflammation, and altered gut microbiota (dysbiosis), play an important role in colic and functional constipation [7,10]. 

The term ‘gut microbiota’ refers to the community of approximately 100 trillion microorganisms that are present in the GI tract. Composition of the gut microbiota can be influenced by many factors. In infants and young children, important factors include gestational age, birth mode, maternal microbiota, exposure to antibiotics, proton pump inhibitors and “biotics” (pro-, pre-, syn-, or post-biotics), and type of feeding [11,12,13]. 

The gut microbiota influences the maturation of the immune system, gut permeability, nutrient absorption, and metabolism, and prevents pathogen colonisation [14]. Several studies and reviews have highlighted the crucial nature of the development of gut microbiota in early life for infant health and its lifelong consequences [15,16].

As measuring gut microbiota is rather invasive, and unethical in subjects who are unable to provide their consent, the faecal microbiome is usually studied instead. Research has shown that faecal microbiota correlates with luminal microbial contents of the large intestine (colon) in terms of species diversity and bacterial abundance [17,18,19].

The aim of this review is to provide a comprehensive, systematic overview of differences in the diversity of the faecal microbiota of infants and young children suffering from FGIDs compared to that of healthy controls. In addition, faecal metabolites and markers of inflammation in relation to faecal microbiota will be reviewed. 

## 2. Methods

The current systematic review followed the preferred reporting items for systematic reviews and meta-analyses (PRISMA) 2009 checklist and is registered in PROSPERO. The registration number is CRD-42020196062.

### 2.1. Search Strategy and Search Terms

Searches of electronic databases were carried out on 5 June 2020. This search was updated on 11 June 2021. Databases searched were Ovid MEDLINE(R), PsycINFO, Web of Science, Cochrane, Scopus, Embase, ScienceDirect, and PubMed 2000 to June 2021. Table 1 shows the search terms that were used.

Furthermore, the reference lists of existing reviews and identified articles were examined individually to supplement the electronic search. A total of 2909 citations were screened against inclusion and exclusion criteria.

### 2.2. Inclusion and Exclusion Criteria

#### 2.2.1. Participants

Studies in children aged 0–5 years suffering from one or more FGIDs of either gender were included. Animal studies were excluded. Studies that did not report results of infants and/or toddlers (0–36 months) separately (e.g., papers reporting on children aged 0–5 years and over, from which results of interest cannot be extracted) were excluded from this review.

#### 2.2.2. Intervention

There were no interventions, although relevant baseline data from intervention studies were included in the data extraction.

#### 2.2.3. Control(s)

Only studies with a healthy (non-FGID) control group were included in this review.

#### 2.2.4. Outcome Measures

This review was limited to studies reporting data on the composition of faecal microbiota. Where available, the following information was included in the review: number of participants and participant characteristics (e.g., age, sex, type of feeding (breast feeding/bottle feeding), mode of delivery (natural birth/c-section), and criteria used for the diagnosis of FGID). In addition, other GI-related measures, including (but not limited to) gut metabolites (e.g., SCFA), bile acids, measures related to gut inflammation (e.g., faecal calprotectin) and gut motility (e.g., ghrelin, motilin), and any reported correlations were included. These outcomes were not a requirement for inclusion but have been extracted where available. 

#### 2.2.5. Design

Study outcomes from observational studies (i.e., cross-sectional, cohort, case-control, and longitudinal studies), as well as baseline data from randomised controlled trials (RCTs), were included in this systematic review.

### 2.3. Study Selection Process

**Figure 1:**
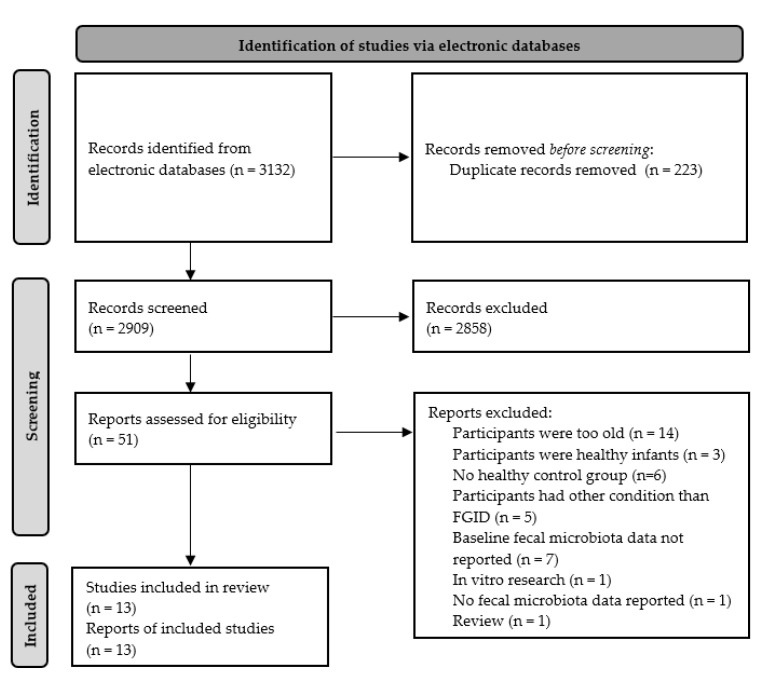
Systematic review flow diagram.

The literature search yielded a total of 3132 citations. Following the removal of duplicates, a total of 2909 citations were retrieved for possible inclusion in the review. The titles and abstracts of these citations were screened by one reviewer (D.H.) to remove obviously irrelevant reports (*n* = 2858), resulting in retention of 51 papers. Another reviewer (U.K.) independently screened the titles and abstracts to establish agreement about the inclusion and exclusion of studies. The initial rate of agreement was 96%. Any disagreements were resolved by discussion, and a consensus decision was reached. The full-text versions of the remaining articles were retrieved and examined for eligibility by both previously mentioned reviewers (D.H. and U.K.) based on the inclusion criteria. Authors were contacted to clarify any missing information. As a result of the screening process, a further 38 articles were excluded. A total of 13 full texts were included in the review (see Figure 1).

### 2.4. Quality Assessment

The quality of all included papers was assessed using the ‘quality assessment tool for reviewing studies with diverse design’ (QATSDD) [20]. Two reviewers (D.H. and U.K.) independently awarded each research paper quality scores by assessing each QATSDD criterion (for example ‘Description of procedure for data collection’) on a 4-point scale from 0 to 3 (0  =  the criterion is not at all described, 1  =  described to some extent, 2  =  moderately described, and 3  =  described in full). The sum of scores of all relevant QATSDD criteria reflects the overall quality of each paper. The scores, expressed as a percentage of the maximum possible score of 42, are included in the data extraction table (Supplementary Table S1).

### 2.5. Data Extraction

The Cochrane data extraction form was modified for the purposes of this review. Data were extracted into the standardised form by one researcher (D.H.), and authors were contacted when insufficient information was provided in the published paper. Data from 50% of these articles were then independently extracted by another researcher (U.K.). Any disagreements were resolved by discussion, and a consensus decision was reached. Extracted data and QAS scores for all papers can be found in Supplementary Table S1.

## 3. Results

### 3.1. Selected Studies

Out of the 13 papers included in this review, the majority assessed the faecal microbiota of colicky infants or associations between the faecal microbiota and fussing/crying behaviour in infants. Only one study studied infants and young children (aged 6–36 months) with functional constipation [21]. Furthermore, one study which primarily focused on the relationship between the microbiome of the first-pass meconium and the development of infantile colic, also reported on associations between the faecal microbiome and other FGIDs, including reflux, constipation, and diarrhoea [22]. No further relevant articles were found assessing faecal microbiota in infants with regurgitation, functional diarrhoea, dyschezia, cyclic vomiting, or rumination syndrome. All papers included a healthy (not FGID) control group, and in two studies, colicky infants or infants with colic-like fussing/crying behaviour were only a small subgroup of the whole study population [22,23]. Two publications reported on RCTs with colicky infants [24,25], from which relevant baseline data were extracted. All relevant study outcomes can be found in Supplementary Table S1 (data extraction table). 

Quality ratings ranged from 45.2% to 70.2% of the maximum score and overall median quality was rated at 56.0%. Papers scored particularly low with respect to detailed recruitment data. In addition, the majority of papers scored low on criteria related to the reporting of statistics: there was no clear evidence of sample size considered in terms of analysis, justification for analytical method selected, or assessment of reliability of the analytical process across publications. In addition, only one of the sequencing studies reported to have corrected for the false-discovery rate (FDR) [26]. Furthermore, whilst most papers had strong discussions in terms of interpretation and implications of the data, they lacked a critical discussion of the strengths and weaknesses of the studies reported.

### 3.2. Confounding Factors That Could Influence Microbiota Composition

There are many potential confounding factors influencing the microbiome composition, such as prematurity, method of delivery, administration of medications such as antibiotics and proton pump inhibitors, and type of feeding (e.g., breastfeeding, formula feeding, other dietary factors, and use of pre, pro-, syn-, or post-biotics). Information on confounding factors was not always reported, but Supplementary Table S2 shows the variance in method of delivery, type of feeding, and use of antibiotics and probiotics within and between studies.

A number of studies included only exclusively breastfed infants [27,28,29,30], one study included only formula-fed infants [31], and the remainder of studies included infants with varying types of feeding (breastfeeding, formula feeding, and/or mixed feeding). In studies that included all types of feeding, the percentage of exclusive breastfeeding was lower in infants with colic than healthy controls [24,32]. In addition, Rhoads et al. reported that exclusively formula-fed infants had a significantly higher alpha diversity, i.e., the distribution of species abundances in a given sample [33], than exclusively breastfed infants. With regards to duration of breastfeeding, however, de Weerth et al. (2013) and Korpela et al. (2020) did not observe a significant difference in breastfeeding duration or age of introduction of milk formula between colicky infants and healthy controls [22,26]. In contrast, infants and young children with functional constipation included in the de Moraes (2016) study had a significantly shorter breastfeeding duration compared to controls. 

The vast majority of research exclusively included term infants, with the exception of one study, in which ~8% of infants and young children in both the FGID and control groups were born prematurely [21].

None of the study populations of the research included in this review were homogenic in terms of mode of delivery (i.e., vaginal delivery or c-section). However, mode of delivery was generally well-matched between infants with a FGID and controls. Only de Weerth et al. (2013) reported percentages of home and hospital births but observed no significant difference between colicky infants and controls [26]. Korpela et al. (2020) looked at associations between the first stool and the development of colic and concluded that delivery mode or exposure to antimicrobials during delivery had no effect on the development of colic [22]. Despite reports of a history of infections and use of antibiotics and probiotics during the first month of life in <20% of colicky infants compared to none of the healthy controls, these observed differences were not found to be statistically significant by Pärtty et al. (2017) [24]. 

Infants and young children with functional constipation were found to have a significantly higher family history of constipation compared to controls [21]. Finally, there were no significant differences in family history of atopy [24,29], order of birth [28], and number of siblings [22] between subjects with colic and subjects without colic. 

### 3.3. Infantile Colic

The majority of publications focusing on faecal microbiota composition in infantile colic obtained measurements at one time point only, with the exception of the RCTs and three studies following colicky infants and healthy controls for a prolonged time from birth onwards, with reported measures at several time points [22,23,26]. De Weert et al. (2013) collected 4 samples during the first month of life, namely at 2 (the meconium sample), 7, 14, and 28 days of age [26]. In addition, another 5 samples were collected at 3 to 5 months of age. Pham et al. (2017) followed a cohort of 40 infants, some of which (*n* = 8, 20%) developed colic. They collected stool samples when the infants were 2 weeks, 1 month, 3 months, and 6 months of age [23]. Finally, Korpela et al. (2020) collected first-pass meconium samples of 212 new-born infants and collected follow-up stool samples at 1 year of age. Moreover, they looked at differences in the first-pass meconium of infants who later developed colic and those who did not and studied associations between faecal microbiota and GI symptoms [22]. 

For the RCTs, only baseline data were extracted as the probiotic interventions studied in these trials would have influenced the faecal microbiota of subjects included, and outcomes do not represent the ‘normal’ faecal microbiota profiles of infants and young children with FGIDs. 

#### 3.3.1. Diagnostic Criteria Used

The included studies used several variations on the definition of infant colic to select their study population. Only one study [22] followed the original Wessel’s criteria, which describes infant colic as excessive crying for more than 3 h per day, for at least 3 days per week, during at least 3 weeks in an otherwise healthy infant [3]. The majority of studies used the modified Wessel’s criteria (see Supplementary Table S1) [24,25,26,27,28,29,30,31,32,34], all of them using less strict criteria, such as crying for more than 3 h per day, for at least 3 days per week, during at least 1 week, which are similar to the Rome III criteria used in one study [23]. Information on crying behaviour was collected based on parental reports (e.g., Barr diary, questionnaires, interviews) in all studies.

#### 3.3.2. Sampling and Storage of Stool Samples

Samples were either collected by parents [26,34], midwives [22], or the study team (research nurses or investigators) [24,27,30,32,35]. In roughly half of the studies, samples were stored at 4–8 °C [23,36] or −20 °C [24,26,31,35,37,38] for a maximum of 24 h until transport to the lab, where samples were usually processed and stored at −80 °C until analysis. In some cases, samples were immediately stored at −80 °C [23,25,30,34] upon collection. Few papers specified the method of transport. De Weerth et al. (2013) reported that, upon parental collection of faecal samples, samples were immediately frozen at −20 °C and then transported in coolers with freezing cartridges/dry ice [26]. 

#### 3.3.3. Method of Analysis of Faecal Microbiota

The majority of included studies analysed collected stool samples using methods based on the 16S rRNA gene (e.g., 16S amplicon sequencing, quantitative polymerase chain reaction (qPCR), or microarrays) [22,23,25,26,27,30,32,34]. However, several papers reported other techniques, including fluorescence in situ hybridisation (FISH) [31], automated ribotyping [29], or even just (Gram) staining and counting bacteria under a microscope [28].

#### 3.3.4. Faecal Microbiota Composition

Reported faecal microbiome composition results have been grouped according to the method of analysis (i.e., 16S sequencing and qPCR vs. other methods) as well as by taxonomy classification (phylum, family, genus, and, where applicable, species). As Table 2 and Table 3 below show, the number of studies reporting on the same families, genera, and/or species within one phylum are limited.

##### qPCR and 16S Sequencing Results

Table 2 presents an overview of the results by NGS and qPCR of faecal microbiota of infants with colic compared to healthy controls. Rhoads et al. (2018) reported a significantly lower abundance of the phylum Actinobacteria as well as the genus *Bifidobacteria* in colicky infants compared to controls [34]. In line with this, de Weerth et al. (2013) observed significantly lower levels of *Bifidobacteria* in infants with colic vs. controls at 1 and 2 weeks of age [26]. In contrast, Savino et al. (2018) found no differences in levels of *Bifidobacteria* in colicky infants compared to healthy controls [25].

Despite Firmicutes being one of the most abundant phyla in colicky infants [34], Korpela et al. (2020) observed a significantly lower relative abundance of Firmicutes and, more specifically, the genus *Lactobacillus* in the first stool of infants who later developed colic. De Weerth et al. (2013) also reported significantly lower levels of *Lactobacilli* in colicky infants compared to controls at 1 and 2 weeks of age. In contrast, another study found significantly higher levels of *Lactobacillus iners* in infants with colic compared to those without [34], and yet another study found no significant differences between colicky infants and healthy controls [25]. In addition, Pham et al. (2017) studied lactate-utilizing bacteria (LUB) in colicky infants and found significantly more non-sulphate-reducing (non-SRB) LUB, such as the Firmicutes *Veillonella* and *Eubacterium hallii* (recently reclassified as *Anaerobutyticum hallii*), as well as a significantly greater non-SRB/SRB ratio in infants with colic compared to controls [23].

The phylum Bacteroidetes was identified to be predominant in colicky infants in several studies [26,27,34], but only one of these—de Weerth et al. (2013)—compared the abundance in the stool of infants with and without colic. The authors reported significantly lower Bacteroidetes in infants with colic during their first two months of life [26].

Rhoads et al. (2018) identified Verrucomicrobia as another predominant phylum in colicky infants. However, the authors reported no significant differences from healthy controls [34].

Finally, de Weerth et al. (2013) found significantly more Proteobacteria in colicky infants than controls at two weeks of age. This was also observed by Rhoads et al. (2018), however, only as a trend. In addition, other studies showed significantly higher levels of the Proteobacteria species *Escherichia coli* [25], *Klebsiella pneumoniae* [32], and genus *Acinetobacter* [34]. In line with these results, Savino et al. (2011) observed significantly higher average counts of coliform bacteria (identified as *Escherichia coli*, *Klebsiella pneumoniae*, *Klebsiella oxytoca*, *Enterobacter aerogenes*, *Enterobacter cloacae*, and *Enterococcus faecalis*) in colicky infants compared to healthy controls.

**Table 2:** Faecal microbiota of infants with colic compared to healthy controls. Results from research using 16S sequencing and/or qPCR.

Phylum	Class	Order	Family	Genus	Species	Studies	Compared to Controls				References
						***n***	**↑**	**=**	**↓**	Unknown *	
Actinobacteria						1			1		[34]
	Actinobacteria	Bifidobacteriales	Bifidobacteriaceae	Bifidobacteria		4		1	2	1	[25,26,27,34]
					*B. Breve*						
					*B. longum*	1				1	[32]
		Coriobacteriales	Coriobacteriaceae	Collinsella		1				1	[27]
Bacteroidetes						3			1	2	[26,27,34]
Firmicutes						2			1	1	[22,34]
	Bacilli	Lactobacillalles	Lactobacillaceae	Lactobacilli		4		1	2	1	[22,25,26,27]
					*L. iners*	1	1				[34]
			Enterococcaceae	Enterococcus		1				1	[27]
			Streptococcaceae	Streptococcus		1				1	[27]
					*S. thermophilus*	1				1	[34]
			Eubacteriaceae	Eubacterium	*E. hallii*	1	1				[23]
				Erysipelatoclostridium		1				1	[27]
	Negativicutes	Vellionellales	Veillonellaceae	Veillonella		2	1			1	[23,27]
Proteobacteria						3	2	1			[26,30,34]
	Gammaproteobateria	Enterobacteriales	Enterobacteriaceae			1	1				[26]
				Escherichia		1				1	[27]
					*E. coli*	2	1			1	[25,32]
				Klebsiella							
					*K. pneumoniae*	1	1				[32]
					*K. oxytoca*						
				Shigella		2				2	[27,32]
				Enterobacter							
					*E. clocae*	1				1	[32]
		Pseudomonadales	Moraxellaceae	Acinetobacter			1				[34]
Verrucomicrobia						1				1	[34]

* No comparison between groups reported.

##### Results Using Other Methods of Analysis

In contrast to results from de Weerth et al. (2013), but in line with their 2018 study [25], Savino et al. (2017), using FISH, reported no significant differences in *Bifidobacteria* in the stools of colicky infants and controls (see Table 3).

In several studies, Savino and colleagues looked at different genera and species of the phylum Firmicutes (see Table 3). With the exception of a significantly lower frequency and mean abundance of *Lactobacilli* observed in a 2004 study [28], they reported no significant differences within the Firmicutes phylum between colicky infants and controls [28,29,31].

Using different methods of analysis, Savino et al. also reported significantly higher levels of the Proteobacteria order Enterobacteriales in infants with colic in 2009 (automated ribotyping) [29], but not 2004 (Gram-staining) [28]. Moreover, they observed significantly increased levels of *Escherichia coli* in colicky infants compared to controls [29]. In line with this, their studies found significantly more Gram-negative bacteria [28] as well as coliform bacteria [29,31] in infants suffering from colic.

**Table 3:** Faecal microbiota of infants with colic compared to healthy controls. Results from research using other methods of analysis.

Phylum	Class	Order	Family	Genus	Species	Studies	Compared to Controls			References
						***n***	**↑**	**=**	**↓**	
Actinobacteria	Actinobacteria	Bifidobacteriales	Bifidobacteriaceae	Bifidobacteria		1		1		[31]
Bacteroidetes						n/a				
Firmicutes	Bacilli	Lactobacillalles	Lactobacillaceae	Lactobacilli		2		1	1	[28,31]
			Enterococcaceae			2		2		[28,31]
				Enterococcus	*E. faecalis*	1		1		[29]
					*E. aerogenes*	1		1		[29]
	Clostridia	Clostridiales	Clotridiaceae	Clostridium		1		1		[28]
Proteobacteria	Gammaproteobateria	Enterobacteriales				2	1	1		[28,29]
				Escherichia						
					*E. coli*	1	1			[29]
				Klebsiella						
					*K. pneumoniae*	1		1		[29]
					*K. oxytoca*	1		1		[29]
				Enterobacter						
					*E. clocae*	1		1		[29]
Verrucomicrobia						n/a				

#### 3.3.5. Microbiota Diversity

In addition to faecal microbiota composition, several papers also reported on the microbiota diversity in infants with colic [23,26,32,34]. Rhoads et al. (2009) observed a significantly lower microbiota diversity in colicky infants compared to controls [32]. In line with this, de Weerth et al. (2013) reported that the microbiota diversity in non-colicky infants increased with time during the first 100 days of life, whereas the microbiota diversity in infants with colic remained low [26]. Moreover, the microbiota diversity (bacterial evenness, i.e., how similar the amounts of the different bacterial groups in the samples are) was significantly lower in infants with colic at postnatal days 14 and 28. However, the bacterial richness (i.e., the number of different species found in the samples) was found to be similar in infants with colic and controls in this study [26], whereas another study reported a significantly higher bacterial richness in infants with colic [34]. Furthermore, in de Weerth’s (2013) study, control infants showed a higher stability of microbiota (at different time points) than did the infants with colic, who had a significantly lower similarity between samples taken at 1 and 2 weeks of age [26]. In line with this, several studies reported an increased individual variability and sample-to-sample variability in colicky infants compared to controls [23,34,36].

#### 3.3.6. Metabolites

The study by Pham et al. (2017) was the only research that reported on levels of lactose, glucose, lactate, and the short-chain fatty acids (SCFAs) acetate, propionate, and butyrate observed in the faecal matter of their study populations [23]. They found no significant difference in metabolomic profiles between colicky and non-colicky infants. However, a colicky infant showed 2-fold higher faecal lactate concentrations at 2 and 3 months of age compared to their non-colicky twin. In contrast, at several time points, high formate and SCFA levels were detected in the faeces of the non-colicky twin, compared to no formate and lower SCFA levels in the colicky twin [23]. Furthermore, in two separate studies, Savino and colleagues studied the gas-forming capability of the microbiota isolated from stool using either a Luria–Bertani broth or a Lauryl sulphate tryptase broth containing lactose as the sole carbon source. They reported that all isolated strains of coliform bacteria from both colicky and healthy infants were found to produce gas from lactose [29,30]. Savino et al. (2017) also studied the pH and ammonia concentration of faecal samples. Results revealed similar pH values in colicky and healthy infants, but significantly higher levels of faecal ammonia in the colicky compared to the healthy group [31]. 

Finally, one study investigated breath hydrogen levels in colicky infants [32]. The authors reported that 50% of their colicky study population vs. 25% of the non-colicky infants had elevated fasting breath hydrogen (H_2_). Moreover, they reported that crying in colicky infants was correlated with high pre-prandial breath hydrogen levels [32].

#### 3.3.7. Markers of Inflammation

Five of the included studies measured markers of inflammation, such as faecal calprotectin, and cytokines and chemokines. Faecal calprotectin was consistently increased in infants with colic. Savino et al. (2018) found significantly higher faecal calprotectin levels in colicky infants compared to controls [24]. In line with this, Rhoads et al. (2009) reported that faecal calprotectin levels of colicky infants were nearly twice those of healthy controls [32]. Moreover, in another study, Rhoads et al. (2018) found faecal calprotectin levels to be the most important factor for classification as colicky or non-colicky infants [34].

Most cytokines, chemokines, and regulatory T cell (Treg) levels in colicky infants were not found to be abnormal or different to healthy controls [25,37,38]. Aparicio et al. (2020) found significantly lower interleukin 7 (IL-7) levels in infants with colic, and Pärtty et al. (2017) reported significantly higher levels of IL8, monocyte chemotactic protein-1 (MCP-1), and macrophage inflammatory protein 1b (MIB-1b) in colicky infants compared to controls [25].

Furthermore, Pärtty et al. (2017) found faecal levels of *Clostridium leptum* to be negatively correlated with proinflammatory markers MCP-1, MIP-1b, and tumour necrosis factor-alpha (TNF-a). In addition, *Clostridium coccoides* was negatively correlated with MCP-1, and *Bifidobacterium breve* levels were positively correlated with chemokine (C-X-C motif) ligand 16 [25].

### 3.4. Constipation

One observational study looked at constipation in infants and young children (aged 6–36 months) [21]. The authors defined constipation as the elimination of hard stools associated with one of the following characteristics: pain or straining while passing stools, scybalous stools, cylindrical and cracked or cylindrical and thick stools, and stool frequency less than three times per week.

#### 3.4.1. Sampling, Storage, and Method of Analysis

Stool samples were placed in sterile polypropylene containers and transported in coolers filled with ice before freezing the samples until analysis. In contrast to studies discussed previously, faecal samples from infants and young children with functional constipation were stored at −18 to −20 °C until DNA extraction and analysis of the samples using qPCR. The primers used for qPCR were selected to identify and quantify *Lactobacillus* spp. and *Bifidobacterium* spp. [21].

#### 3.4.2. Faecal Microbiota Composition

De Moraes et al. reported that constipated children had a lower concentration of *Lactobacillus* per milligram of stool than non-constipated children, but there was no difference in *Bifidobacterium* levels [21].

### 3.5. Other FGID

In addition to reporting on associations between the first-pass meconium and the development of infantile colic, Korpela et al. (2020) studied differences in the microbiome of the first-pass meconium of infants who later developed reflux, constipation, or diarrhoea, and those who did not [22]. No major differences in the main phyla and genera, number of OTUs, or bacterial diversity were observed between infants with FGID and healthy controls. Similarly, the authors did not report any significant differences between the faecal microbiome at 12 months of age in the infants who developed reflux or constipation. However, they did observe a significant difference in the 12-month stool sample of the infants with parental-reported diarrhoea compared to healthy controls. The relative abundance of the genus *Lactobacillus* was higher in those who had suffered from diarrhoea [22]. 

## 4. Discussion

### 4.1. Infantile Colic

#### 4.1.1. Faecal Microbiota and Metabolites

Despite the growing evidence for the pivotal role that microbiota plays in health and disease, there is lack of an accurate description of a ‘normal’ or ‘healthy’ microbiota [39], dysbiosis, or the establishment of the gut microbiome in neonates [40]. However, the current consensus is that one of the primary factors influencing establishment of the gut microbiota is the type of feeding, i.e., breastfeeding vs. formula feeding [13], which may already account for some of the differences observed between studies included in this review. It is generally known that several breastmilk components function as anti-microbial agents, suppressing gut microbial diversity, possibly to prevent pathogen colonisation [13,41,42].

Research suggests that anaerobic bacteria, primarily *Staphylococcus*, *Streptococcus,* and *Enterococcus* spp. (Firmicutes), as well as *Enterobacteriaceae* spp. (Proteobacteria) and *Bifidobacterium* spp. (Actinobacteria), act as pioneer bacteria, reaching high counts within the infant gut in the first days and weeks of life [39,43]. These pioneer bacteria are followed by other members of the Firmicutes (e.g., *Lactobacilli*) early in life [39]. Data on the onset of other anaerobes such as *Bacteroides* and their population levels are inconsistent, with some studies suggesting an early onset whilst other studies suggest that these species do not establish themselves in the gut until an infant reaches 12 months of age [43].

Results included in this review suggest a dysbiosis, defined as a different (faecal) microbiota from the healthy infant, among these pioneering bacteria in colicky infants. Using next-generation sequencing techniques, studies included in this review generally reported significantly lower levels of several early colonisers in colicky infants compared to healthy controls, namely those from the phyla Firmicutes (*Lactobacilli*), Bacteroidetes, and Actinobacteria (*Bifidobacteria*) [22,26,27,34], with the exception of two studies: one study found significantly higher levels of *Lactobacillus iners* [34], and another study found significantly higher levels of *Veillonella* and *Eubacterium hallii* [23], all three belonging to the phylum Firmicutes.

In contrast, the phylum Proteobacteria was consistently found to be increased in colicky infants [26,28,29,31], especially in their first two weeks of life [26]. In line with this, several studies included in this review found a significant increase in Proteobacteria in colicky infants at the level of: family *Enterobacteriaceae* [26], genus *Acinetobacter* [34], and species *Escherichia coli* [25,32] and *Klebsiella pneumoniae* [32].

In research using other methods of analysis of faecal microbiota, including microscopic examination, FISH, and automated ribotyping, the majority of these observed differences in pioneering bacteria were not reported [28,29,31], with a few exceptions: First, using morphological characterisation and microscopic examination, one study reported significantly lower levels of *Lactobacilli* in colicky infants compared to controls [28]. Second, using automated ribotyping, Savino et al. (2009) reported significantly higher levels of *Escherichia coli,* a species belonging to the Proteobacteria [29]. Furthermore, in line with the observed increase in Proteobacteria, studies using Gram-staining found significantly more Gram-negative bacteria [28] as well as coliform bacteria [29,31] in infants suffering from colic.

In addition to alterations in the composition of faecal microbiota, several papers reported significantly lower microbiota diversity as well as stability in colicky infants compared to controls. Microbial diversity leads to functional redundancy and metabolic flexibility, and therefore resilience of the microbe–microbe and host–microbe interactions [44]. 

Information about microbial community taxonomic composition alone does not necessarily provide understanding of the community’s function. Functional characterisation of the microbiome’s metabolites might be even more important for predicting health and disease. Moreover, in addition to elucidating on the microbiome’s function, faecal metabolites may provide a good marker of the gut microbiome. A recent paper showed that the faecal metabolome largely reflects gut microbial composition, explaining on average 67.7% (±18.8%) of its variance [45].

Only a limited number of studies included in this review evaluated microbial metabolites. Primary colonisers of the infant gut (i.e., *Lactobacillus, Bifidobacterium, Bacteroides, Streptococcus, Staphylococcus,* and *Enterococcus*) are lactate-producing bacteria (LPB) [40]. Lactate produced by these bacteria is in turn used by SRB-LUB species, such as *Veillonella* and *Eubacterium hallii,* which were found to be significantly increased in colicky infants [23]. Both bacteria, belonging to the phylum Firmicutes, produce H_2_ when utilizing lactate. The observed increase in these H_2_-producing bacteria is in line with the observation that a subset of colicky infants in a study included in this review had elevated pre-prandial breath H_2_ levels [32]. In another study, which did not include a control group, it was also reported that 25% (of *n* = 20) of infants with colic had elevated fasting breath hydrogen [37]. Excess H_2_ production from lactate utilisation may be responsible for the high incidence of acute bloating and cramping in early life. In addition to producing H_2_, *Eubacterium hallii* produces butyrate, which is involved in gut tissue development and maturation, modulation of colon motility, immune modulation, oxidative stress reduction, and diarrhoea control [46,47]. Despite showing increased levels of *Eubacterium hallii*, as well as its metabolite H_2_, butyrate levels were not found to be increased in the same sample of infants [23]. This might indicate a difference in substrate availability, i.e., *Eubacterium hallii* might produce different metabolites based on the type(s) of substrates available.

Finally, Pham et al. (2017) reported a significant difference in the SRB-LUB and non-SRB-LUB in colicky infants. Increased numbers of SRB could result in elevated hydrogen sulphide (H_2_S) levels, which could lead to GI discomfort. Based on all of the above, the authors concluded that the metabolic impact of microbiota on gut health could be mediated by the accumulation of either lactate or other end products of the lactate utilisation process, such as H_2_ or H_2_S. However, they found no differences in levels of lactate or butyrate between colicky infants and healthy controls [23].

A more comprehensive analysis of bacterial metabolites with a focus on SCFAs would be beneficial to our understanding of these complex interactions. Metabolites such as H_2_S and bile acid derivatives may exacerbate inflammatory states, while changes in SCFA production may influence epithelial gut barrier integrity [48]. It could also provide an explanation for the postulated role of the nervous system in the pathophysiology of colic [49], as accumulating evidence points to a critical role for the gut microbiome in regulating the gut–brain axis. For example, butyrate, apart from being the main source of energy for colonocytes [50], has been shown to influence the release of the neurotransmitter serotonin [51]. Serotonin, in turn, might lead to infant colic by affecting gastrointestinal motility, pain conduction, and pain sensation [52]. Higher levels of serotonin were found in infants with colic than in healthy infants [53]. In addition, propionate produced by gut bacteria protects the blood–brain barrier (BBB) from oxidative stress [54] and an increase in its production has been associated with reduced stress behaviours in mice [55]. SCFAs can also affect inflammation by modulating the production and recruitment of immune cells, such as T cells, neutrophils, and inflammatory cytokines [56].

#### 4.1.2. Markers of Inflammation

Faecal calprotectin, a measure of inflammation, was consistently increased in infants with colic compared to controls. Two studies without healthy controls reported that faecal calprotectin levels of infants with colic were above the normal clinical range in adults [37,38]. However, faecal calprotectin levels of healthy infants and young children have been shown to be higher than those of adults and, to date, no paediatric reference range of faecal calprotectin has been established [57]. Other markers of inflammation were less frequently studied. Only one out of three studies reported significantly higher levels of certain chemokines (IL8, MCP1, MIB-1b). Moreover, the authors reported several associations between inflammatory markers, faecal microbiota, and crying. Based on their findings, they concluded that, in addition to gut microbiota alterations, infantile colic is associated with low-grade systemic inflammation.

### 4.2. Functional Constipation

Only one study included in this review reported on faecal microbiota in infants and toddlers with functional constipation [21]. The authors limited their analysis of faecal microbiota to levels of *Bifidobacteria* and *Lactobacilli.* Compared to healthy controls, infants and toddlers with constipation had significantly lower levels of *Lactobacilli* in their stools. Moreover, inflammatory markers and bacterial metabolites were not assessed in this study. Overall, based on this review, it seems that the role of microbiota and their metabolites in the development and course of paediatric functional constipation is severely understudied.

### 4.3. Limitations of Included Studies

#### 4.3.1. Study Population Selection

The majority of studies included in this review reported limited information with regards to well-known confounding factors that influence the composition of the microbiome. For example, only three studies included information with regards to the history of use of antibiotics in their samples, whilst the impact of antibiotic and probiotic use on microbiota is well-documented [11,58,59,60]. In addition, not all papers reported on the mode of delivery or gestational age of subjects included in the study. Furthermore, several of the studies included in this review were not homogenic in terms of subject characteristics, such as type of feeding, or type of delivery. However, in studies including a healthy control group, colicky and non-colicky infants were generally well-matched with regards to these potentially confounding factors. Finally, the sample size of some of the studies included in this review was relatively small, varying between 8 [23] and 60 [25] subjects (median 37, see Supplementary Table S1 for sample sizes of all individual studies). As there is substantial variability in gut, and therefore faecal, microbiota diversity amongst individuals, and especially in infancy, it is difficult to obtain significant results. Research with larger samples sizes may help to elucidate alterations in the microbiota of infants and young children with FGID.

#### 4.3.2. Stool Sampling, Storage, and Processing

There are no universally accepted standards for the collection and microbial analysis of stool samples [61]. This is also apparent when looking at the methodology of the papers included in this review. The majority of papers did not report their method of stool sampling, processing, and DNA extraction in detail. However, the methods of sampling, processing, and storage can have a big impact on the detection and quantification of bacteria [61].

In addition, none of the reported studies collected information on the appearance and density of the collected stool samples (e.g., Bristol Stool chart), which reflect the colonic transit time and could help interpret the data and explain differences observed between different studies. Faecal microbiota do not necessarily represent gut microbiota, but might rather depend on transit time [62,63]. For example, Vandeputte et al. (2016) found that stool consistency was strongly associated with gut microbiota richness and composition, enterotypes, and bacterial growth rates [62].

#### 4.3.3. Analysis of Stool Samples

The use of different methods, as well as differences in the choice of which variable regions are assessed, complicates the comparison and interpretation of results of the different papers included in this review. Moreover, the different methodologies used could be the reason that some of the reported results are inconsistent.

#### 4.3.4. Reported Outcome Measures

Overall, only a few studies reported on the same genera or species of bacteria, resulting in limited data with regards to independent genera or species. Moreover, for some studies, the differences, or lack thereof, between colicky infants and their healthy controls were not always clearly reported [27,32,34].

In addition, faecal metabolites were only assessed in 5 of the 13 studies included in this review. Of these, only one paper reported on SCFA [23]. Furthermore, two of these studies, as well as three other papers, reported on markers of inflammation. As discussed before, including metabolites and inflammatory markers when assessing microbial composition would help shed light on the functionality and interactions of the microbiota and its by-products.

Finally, the literature search of this systematic review only captured one study which reported on the associations between faecal microbiota and the development of reflux and diarrhoea in infants [22]. This paper reported lower levels of *Lactobacilli* in the stool of 12-month-old infants with a history of diarrhoea compared to controls. Moreover, no papers studied the potential role of faecal microbiota in cyclic vomiting syndrome, even though some experts view cyclic vomiting syndrome as a gut–brain disorder and gut microbiota might well play a role in its pathology [64]. The intestine harbours the highest density of microbiota [65] but is not the only ecological niche populated by microbiome within infants. The oral cavity, nares, oesophagus, and skin are also important habitats [65]. It is plausible that changes in microbiota during the FGIDs may not be limited to microorganisms in the gut only [39]. For example, a study reported several differences in the salivary microbiome of untreated adult GERD patients compared to healthy controls [66]. Similarly, a more acidic oesophageal environment observed in infants with regurgitation may also favour a different microbiome. Oral microbiota can spread throughout the body and has been associated with a number of diseases [67]. Additionally, diet and geographic location also appear to affect the oesophageal microbiome (as well as that of the oral cavity, upper respiratory tract, and even the skin) [65], offering opportunities to modulate it. Therefore, in the future, studies aiming to unravel the relation between the microbiome and FGIDs, and especially regurgitation, should consider broadening the scope to other less-explored microbiota populations.

### 4.4. Limitations of This Review

This review was limited to papers that included measures of faecal microbiota. As a result, research in infants and young children with FGIDs and their controls, which studied other (e.g., oral, oesophageal) microbiota, metabolites, and/or inflammatory markers, was missed. These outcomes are likely to shed more light on the pathophysiology of FGIDs.

## 5. Conclusions

Overall, research with regards to (changes in) the faecal microbiota of infants with symptoms of colic is inconsistent, and there is a lack of homogeneity in the inclusion of study subjects and outcome measures (e.g., specific bacterial species studied, faecal metabolites, inflammatory markers, but also confounding factors) as well as analytical measures used. In addition, it is unclear whether infantile colic is the result of observed differences in the microbiome, or whether changes in the microbiota cause FGIDs.

Nonetheless, data show alterations in microbial diversity, stability, and colonisation patterns. Moreover, several studies (eight) reported an increase in species of (pathogenic) Proteobacteria, and some studies (six) reported a decrease in (beneficial) bacteria such as Lactobacilli and Bifidobacteria. It should be noted that only nine studies assessed Proteobacteria, and not all studies reported on levels of Bifidobacteria or Lactobacilli in colicky infants compared to controls (four and six studies, respectively). In addition to dysbiosis, accumulation of lactate, H_2_, and/or H_2_S, as well as low-grade inflammation, might all play a role in the pathophysiology of infantile colic.

With regards to other FGIDs, insights on microbiota are limited or lacking, and more research is needed to unravel the underlying causes of these disorders.

Finally, there is a need for more standardised methods within research of faecal microbiota in FGIDs, including but not limited to the inclusion of other microbiota, faecal metabolites, and inflammatory markers to obtain a more comprehensive picture and understanding of infant and childhood FGIDs.

## Figures and Tables

**Table 1:** Search terms used in electronic databases.

Search Terms	AND/OR
“microbio *” OR “dysbiosis” OR “Bifido *” OR “Lactobacill *” OR “Proteobacter *” OR “Escherichia” OR “Klebseilla” OR “Bacteroidetes” OR “Klebsiella” OR “Serratia” OR “Vibrio” OR “Yersinia” OR “Pseudomonas” OR “Enterobacter *” OR “bacteria”	
“functional gastrointestinal disorder *” OR “FGID *” OR “colic” OR “reflux” OR “regurgitat *” OR “GER *” OR “constipation” OR “diarrhoea *” OR “diarrhoea *” OR “vomiting” OR “dyschezia” OR “rumination” OR “inflammatory bowel dis *” OR “IBS”	AND
“infant *” OR “neonat *” OR “toddler *” OR “child *” OR “paediatric” OR “paediatric” OR “newborn” OR “baby” OR “babies”	AND

* truncation.

## Data Availability

Not applicable.

## Supplementary Materials

The following supporting information can be downloaded at: https://www.mdpi.com/article/10.3390/nu14050974/s1, Table S1: Data extraction table; Table S2: Demographics of participants in the studies included in this systematic review.
